# Discovery of a *mcr-1-*bearing plasmid in commensal colistin-resistant *Escherichia coli* from healthy broilers in Faisalabad, Pakistan

**DOI:** 10.1080/21505594.2018.1462060

**Published:** 2018-06-21

**Authors:** Jiali Lv, Mashkoor Mohsin, Sheng Lei, Swaminath Srinivas, Raja Talish Wiqar, Jingxia Lin, Youjun Feng

**Affiliations:** a School of Food and Biological Engineering, Shaanxi University of Science and Technology, Xi'an, Shaanxi, China; b Institute of Microbiology, University of Agriculture, Faisalabad, Pakistan; c Department of Medical Microbiology and Parasitology, Zhejiang University School of Medicine, Hangzhou, Zhejiang, China; d Department of Biochemistry, University of Illinois, Urbana, USA; e College of Animal Sciences, Zhejiang University, Hangzhou, Zhejiang, China

**Keywords:** colistin resistance, enteric bacteria, gut microbiome, IncI2, Lipid A, *mcr-1*, Pakistan, poultry

## Letter to the editor

Polymyxins, like colistin (polymyxin E), are a group of cationic antimicrobial cyclic polypeptide, which have been extensively-used as prophylactic feed additives in animal production since the 1960s [1]. A strong association has especially been drawn between antimicrobial use and resistance in poultry and pig farms [2–5]. The advent and rise of multi-drug resistant bacteria has now prompted the re-introduction of colistin as a last-resort treatment option in human medicine [6,7]. However, the recent emergence and diversity of plasmid-borne mobile colistin resistance determinants (*mcr-1* to *mcr-5*) in *Enterobacteriaceae* has severely challenged its use in a clinical setting [8–12]. In the past two years, *mcr-1* has been detected in over 40 countries across 5 of 7 continents worldwide [13–15]. Except rare cases of chromosomally-integrated *mcr-1* [16,17], its prevalent transmission relies on the transfer by diversified plasmids of different replication incompatibilities [18,19].

Mechanistically, the *mcr-1* gene that encodes phosphoethanolamine transferase mediates the modification of the lipopolysaccharide layer (LPS) of the outer membrane of Gram-negative bacteria through the addition of phosphoethanolamine (PEA) to the 1 (or 4′)-phosphate position of lipid A moieties [20,21]. This reduces the affinity of polymyxin antibiotics to their primary target, the LPS layer. Since its discovery during routine surveillance in China [8], increasingly-accumulated evidence suggested the presence of *mcr-1*-bearing bacteria in food producing animals and humans across the world. To the best of our knowledge, the leading two types of *mcr-1-*harboring plasmids referred to IncI2 [8,18,22,23] and IncX4 [24–26], have greatly facilitated global dissemination of *mcr-1* colistin resistance [14,27]. In Pakistan, the *mcr-1* gene has been identified in *Escherichia coli* (*E. coli*) isolates from human [28], wildlife [29] and a broiler suffering from colibacillosis [30]. However, little is known about the prevalence of *mcr-1* and its genetic environment in commensal *E. coli* isolates from poultry in Pakistan.

**Figure 1.**
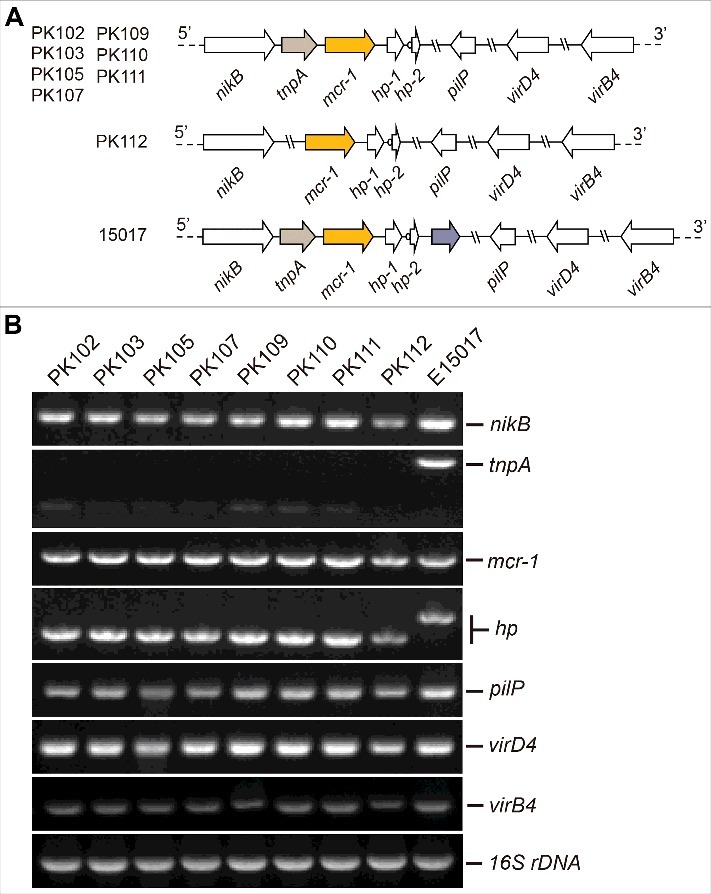
Genetic analyses of *mcr-1*-harboring plasmids in this study A. Scheme of different *mcr-1*-bearing plasmids B. PCR assays of *mcr-1* and neighboring loci in plasmids 16S rDNA is specific to the *E. coli* specie.

From December 2016 to January 2017, cloacal swabs from a total of 100 healthy broiler chicken were obtained from four commercial farms (n = 25 each) in the Faisalabad region of Pakistan. To screen the colistin resistant *E. coli*, all the samples were seeded directly onto MacConkey agar supplemented with 4 μg/ml of colistin and were incubated at 37°C for 24 hours. Of 100 birds, colistin resistant *E. coli* were found in only 8 (8%) samples. A single colony of *E. coli* was selected per sample and identified using API 20E biochemical strips (bioMérieux, Marcy l'Etoile, France). The presence of *mcr-1* gene was confirmed among all 8 *E. coli* isolates by conventional PCR as we recently conducted [19,31]. Subsequently, the minimum inhibitory concentration (MIC) of colistin among these strains was tested by micro-dilution according to the guidelines of Clinical and Laboratory Standards Institute [32,33]. The *mcr-1*-positive *E. coli* gave MIC of colistin between 2–8 μg/ml (Table 1). Plasmids were extracted from *mcr-1-*positive *E. coli* using alkaline lysis method. To elucidate the genetic context of *mcr-1* on these plasmids, the conventional multiplex PCR with 7 primer sets was performed (Table S1) as we recently described [3,19]. The plasmids isolated from the different strains had unexpectedly similar PCR profiles with the exception of pPK112 which lacks the *tnpA* loci (Fig. 1B). Genetic context of *mcr-1* shows that all the plasmids lack the insertion element IS*Apl*1 (Fig. 1A), which has been responsible for insertion of *mcr-1* in previous studies [34]. Also, these plasmids (Fig. 1A) are identical to the *mcr-1*-carrying plasmid pE15017 isolated in China [19,22]. These bacteria were subjected to multi-locus sequence typing analyses with the Warwick method (http://mlst.warwick.ac.uk/mlst/). Diversified sequence types were detected, namely, ST10, ST2847, ST155, ST361 and ST6395. Evidently, all strains belonged to different STs with the exception of two strains of ST361 (Table 1). Despite that none of these STs have been reported from Pakistan in the past, ST10 and ST155 have been reported in *mcr-1*-harboring *E. coli* isolated from chicken in China [17,35].

**Table 1.** Characteristics of *mcr-1*-positive *E. coli* isolates from healthy broilers in Faisalabad, Pakistan.

Strains	Source	Date	MIC (μg/ml)	MLST	Farm no.
PK102	cloacal	27/12/2016	≥8	ST10	1
PK103	cloacal	27/12/2016	≥4	ST2847	1
PK105	cloacal	27/12/2016	≥8	ST155	1
PK107	cloacal	27/12/2016	≥8	New ST	1
PK109	cloacal	27/12/2016	≥4	ST361	1
PK110	cloacal	30/01/2017	≥4	ST6395	3
PK111	cloacal	30/01/2017	≥2	ST361	3
PK112	cloacal	30/01/2017	≥8	New ST	3

**Figure 2.**
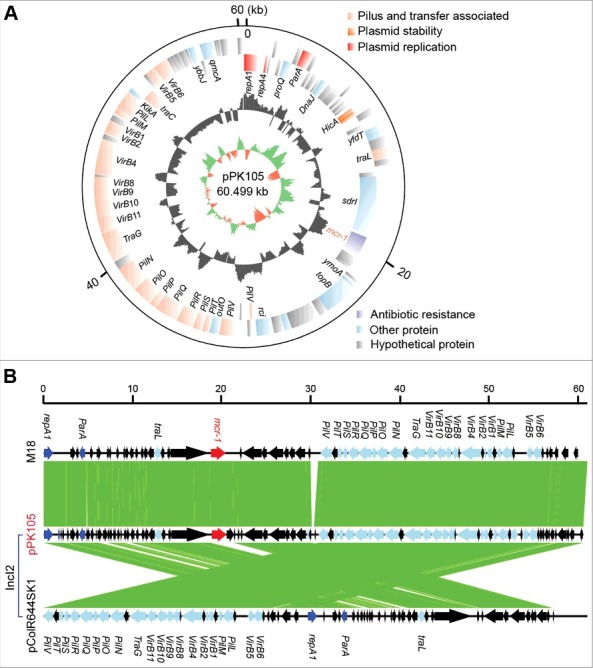
The representative *mcr-1*-bearing plasmid in a colistin resistant *E. coli* isolate PK105 A. Diagram for the *mcr-1*-positive plasmid (pPK105) that exists in the E. coli isolate PK105 B. Colinear comparison of the IncI2-type plasmid pPK105 with two closely-related plasmids *mcr-1*-postive M18 and *mcr-1*-negative pColR644SK1.

Subsequently, a representative *mcr-1-*carrying plasmid pPK105 (Table 1) was subjected to whole genome sequencing using the method of Illumina HiSeq X-ten. The plasmid sequences were annotated by RAST, and the genome maps were drawn with the Circos program. As a result, the genome size of pPK105 was determined to be 60.499 kb (Acc. no.: MG808035, Fig. 2A), encoding hundreds of open reading frames with a GC content of 42.3%. Unlike the prevalent IncX4 type plasmid reported in Pakistan, Plasmid Finder at the web server (https://cge.cbs.dtu.dk/services/PlasmidFinder/) indicated that pPK105 is a member of IncI2-type plasmid family (Fig. 2B). Notably, *mcr-1* is the only resistance gene detected in pPK105 (Fig. 2B). It is quite different from the scenarios observed in the other *mcr-1*-containing Pakistan isolate coharboring ESBL (extended-spectrum β-lactamase gene) and heavy-metal resistance. Although that IS*Apl1* sequence is missing, pPK105 retains the two conjugative genes *vir* and *pil* (Fig. 2). Intriguingly, we noticed that the *mcr-1* gene in pPK105 is next to a *sdrl* gene, a serine-aspartate repeat surface protein known to bind collagen (Fig. 2B). However, its relevance remains unclear. Nevertheless, it has been shown earlier that IS*Apl1* transposon element is highly unstable in IncI2 plasmid [36].

**Figure 3.**
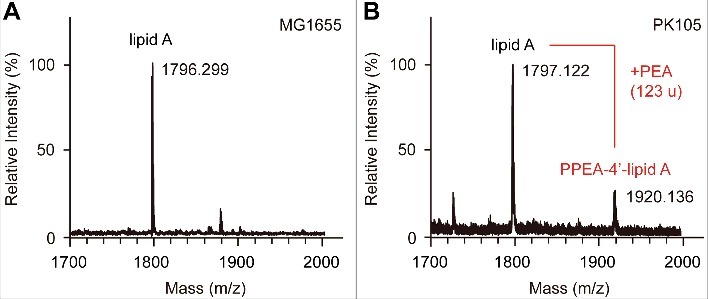
MALDI-TOF MS analyses of lipid A pools of *E. coli* strains with (or without) *mcr-1* A. MS profile of the LPS-lipid A in the negative control strain *E. coli* MG1655 B. MS spectrum of the LPS-lipid A in PK105, a representative strain of *E. coli* carrying *mcr-1.* A single modification may occur at the 1 (or 4′) position. The position indicated here is suggestive [20].

For further investigation, the chemical modification of lipid A by the *mcr-1* protein product MCR-1, the bacterial LPS was extracted as we conducted earlier [20,37] and then subjected to matrix-assisted laser desorption ionization–time of flight mass spectrometry (MALDI-TOF MS) [38]. In comparison to *E. coli* MG1655 (m/z 1796.29, Fig. 3A), a shift in the predominant lipid A species (m/z 1920.136, Fig. 3A) was observed in the *mcr-1*-expressing PK105 strain (Δ_mass_ is close to 123) corresponding to the addition of a PEA moiety (Fig. 3B). This new peak corresponds to a single modification that may occur at either the 1 or the 4′ position (Fig. 3B). This highlighted that surface remodeling by the *mcr-1*-encoding phosphoethanolamine transferase contributes to the resultant colistin resistance [14].

The discovery of the *mcr-1* gene, prompted a shift in focus from chromosomal mutations causing colistin-resistance to a transmissible plasmid-borne colistin resistance determinant. In addition to clinical isolates in humans, antimicrobial surveillance programs in Europe have identified *mcr-1* in commensal bacterial populations from broilers, pigs and turkeys [39]. This study shows a similar threatening scenario in the Faisalabad region of Pakistan where high rates of *mcr-1* positive *E. coli* were identified in healthy broilers. Retrospectively, the discovery of diverse clonal backgrounds of *E. coli* harboring the plasmid-borne *mcr-1* is similar to scenarios observed earlier in Chinese poultry.

Similarly, data on global population structure of *mcr-1-*positive *E. coli* showed large diversity in STs but limited plasmid types, particularly with regional spread of IncHI2 plasmids in Europe and IncI2 in Asia [14]. This indicated the possible spread of a single *mcr-1* colistin resistance gene across large geographical distances. It seems likely that farm animals act as a reservoir for the genetic diversity of *mcr-1* [40].

## Supplementary Material

1462060.zip
