# Supplementary material for: Discovery of a mcr-1-bearing plasmid in commensal colistin-resistant Escherichia coli from healthy broilers in Faisalabad, Pakistan
Source: Virulence. 2018 Jun 21;9(1):994–9. doi: 10.1080/21505594.2018.1462060 (PMC6037438; doi:10.1080/21505594.2018.1462060)
Supplement: 1462060.zip [file kvir-09-01-1462060-s001.zip › 1462060/2018_4_2_Virulence_SI_rev.doc]

**Supplemental Information**

**Supplemental Methods**

**Determination of Colistin Susceptibility**

To quantify the minimum inhibitory concentration (MIC) of colistin in different *mcr-1*-bearing strains, liquid broth dilution tests were carried out as recommended by EUCAST with cation-adjusted Mueller-Hinton Broth (CAMHB) . Briefly, overnight cultures were diluted 100-fold in fresh CAMHB medium and grown until OD600 of 0.5. To measure MIC value, these cultures were diluted again to OD600 0.05 in CAMHB containing varying level of colistin (ranging from 0, 0.25, 0.5, 1.0, 2.0, 4.0, 8.0, 16.0 to 32.0 μg/ml). In addition, bacterial viabilities of *mcr-1*-carring *E. coli* were also confirmed on LBA plates with colistin in series of dilution as suggested by Clinical and Laboratory Standards Institute (CLSI) .

**PCR and MLST Analyses**

The plasmid-type of *mcr*-1-harbouring plasmids were detected using PCR assays coupled with Sanger sequencing as we recently established . The specific primers used in PCR assays are indicated in **Table S1**. Multi-locus sequence typing (MLST) was used to analyze all clinical isolates. The procedure and gene specific primers (**Table S1**) for MLST were obtained from the website (<http://enterobase.warwick.ac.uk/species/ecoli/warwick_mlst_legacy>). Sequences of seven housekeeping genes were uploaded on the website (<http://enterobase.warwick.ac.uk/species/ecoli/warwick_mlst_legacy>) to acquire the specific MLST type of isolates.

**Genome Sequencing and Assembly**

Sequencing libraries of the plasmid was sequenced on an Illumina HiSeq X-ten platform (Illumina, USA). Subsequently SPAdes (version 3.11.0) was used to assemble the sequence with a k-mer based approach and select the optimal assembly results. In the end, PCR amplification and Sanger sequencing were used to complete plasmid splicing. RAST software was used to predict functional genes in the plasmid. RAST prediction allows us to obtain the classification and location information of functional genes within plasmid sequence. Finally, Circos (version 0.69) software was used to draw the complete plasmid.

**Extraction and Purification of Lipid A**

The crude lipid A was obtained as described earlier . The purified lipid A was judged with silver staining and then subjected to further analyses of MALDI-TOF MS .

**Supplementary Tables**

**Table S1** Primers used in this study

| Primers | Primer sequence |
| --- | --- |
| *nikB*-F* | 5'-GAT GAA CTT GAT CAT CGT GTT GT-3' |
| *nikB*-R* | 5'-GTA ATT CTG ACG AAA AAG AGG A-3' |
| *tnpA*-F* | 5'-GGT TTT CGG GCT TTT TAA GAG-3' |
| *tnpA*-R* | 5'-TAG CAC ATA GCG ATA CGA TG-3' |
| *mcr-*1-F* | 5'-ATG ATG CAG CAT ACT TCT GTG-3' |
| *mcr*-1-R* | 5'-TCA GCG GAT GAA TGC GGT G-3' |
| *hp*-F* | 5'-GAT AAG CAA ACT GGC ATC ACG-3' |
| *hp*-R* | 5'-GAA CCC TGT ATA TAG CCT GTC-3' |
| *pilP*-F* | 5'-TTA AAG AAT AAG CTG GCG TTT C-3' |
| *pilP*-R* | 5'-ATG TTA AAA ATA ATT AAA CCA ACG-3' |
| *virD4*-F* | 5'-AAT GTC AAC ATG ATT GTT AC-3' |
| *virD4*-R* | 5'-GAA CAT AAC CCG GAC CTG AAA T-3' |
| *virB4*-F* | 5'-AAC TCT TTT TCA GTA AGC CCA AT-3' |
| *virB4*-R* | 5'-TTA ATG TTT GTT GTG GAT TAC AAC C-3' |
| *E16S*-F* | 5'-AAA TTG AAG AGT TTG ATC ATG G-3' |
| *E16S*-R* | 5'-GCT TCT TTA AGG TAA GGA GGT-3' |
| *adk*-F | 5'-ATT CTG CTT GGC GCT CCG GG-3' |
| *adk*-R | 5'-CCG TCA ACT TTC GCG TAT TT-3' |
| *fumC*-F | 5'-TCA CAG GTC GCC AGC GCT TC-3' |
| *fumC*-R | 5'-GTA CGC AGC GAA AAA GAT TC-3' |
| *gyrB*-F | 5'-TCG GCG ACA CGG ATG ACG GC-3' |
| *gyrB*-R | 5'-ATC AGG CCT TCA CGC GCA TC-3' |
| *icd*-F | 5'-ATG GAA AGT AAA GTA GTT GTT CCG GCA CA-3' |
| *icd*-R | 5'-GGA CGC AGC AGG ATC TGT T-3' |
| *mdh*-F | 5'-ATG AAA GTC GCA GTC CTC GGC GCT GCT GGC GG-3' |
| *mdh*-R | 5'-TTA ACG AAC TCC TGC CCC AGA GCG ATA TCT TTC TT-3' |
| *purA*-F | 5'-CGC GCT GAT GAA AGA GAT GA-3' |
| *purA*-R | 5'-CAT ACG GTA AGC CAC GCA GA-3' |
| *recA*-F | 5'-CGC ATT CGC TTT ACC CTG ACC-3' |
| *recA*-R | 5'-TCG TCG AAA TCT ACG GAC CGG A-3' |

*The primers used in PCR assays for plasmid typing are from Ye *et al*. described earlier. The expected sizes of PCR amplicons are as follows: 705 bp for *nikB*; 1504 bp for *tnpA*; 1626 bp for *mcr-1*; 1646 bp for *hp*; 495 bp for *pilP*; 552 bp for *virD4*; 780 bp for *virB4*; and 1554 bp for *E16S*, respectively.

**Supplemental references**

1.	Matuschek E, Brown DF, Kahlmeter G. 2014. Development of the EUCAST disk diffusion antimicrobial susceptibility testing method and its implementation in routine microbiology laboratories. Clin Microbiol Infect 20:O255-66.

2.	Brown D, Canton R, Dubreuil L, Gatermann S, Giske C, MacGowan A, Martinez-Martinez L, Mouton J, Skov R, Steinbakk M, Walton C, Heuer O, Struelens MJ, Diaz Hogberg L, Kahlmeter G. 2015. Widespread implementation of EUCAST breakpoints for antibacterial susceptibility testing in Europe. Euro Surveill 20.

3.	Bakthavatchalam YD, Veeraraghavan B. 2017. Challenges, Issues and Warnings from CLSI and EUCAST Working Group on Polymyxin Susceptibility Testing. J Clin Diagn Res 11:DL03-DL04.

4.	Galani I, Kontopidou F, Souli M, Rekatsina PD, Koratzanis E, Deliolanis J, Giamarellou H. 2008. Colistin susceptibility testing by Etest and disk diffusion methods. Int J Antimicrob Agents 31:434-9.

5.	Ye H, Li Y, Li Z, Gao R, Zhang H, Wen R, Gao GF, Hu Q, Feng Y. 2016. Diversified *mcr-1*-harbouring plasmid reservoirs confer resistance to colistin in human gut microbiota. mBio 7:e00177.

6.	Li Z, Tan C, Lin J, Feng Y. 2016. Diversified variants of the *mcr-1*-carrying plasmid reservoir in the swine lung microbiota. Sci China Life Sci 59:971-3.

7.	Gao R, Wang Q, Li P, Li Z, Feng Y. 2016. Genome sequence and characteristics of plasmid pWH12, a variant of the *mcr-1*-harbouring plasmid pHNSHP45, from the multi-drug resistant *E. coli*. Virulence 7:732-5.

8.	Sun J, Xu Y, Gao R, Lin J, Wei W, Srinivas S, Li D, Yang RS, Li XP, Liao XP, Liu YH, Feng Y. 2017. Deciphering MCR-2 colistin resistance. mBio 8:pii: e00625-17.

9.	Xu Y, Lin J, Cui T, Srinivas S, Feng Y. 2018. Mechanistic insights into transferable polymyxin resistance among gut bacteria. J Biol Chem 293:4350-4365.

10.	Gao R, Hu Y, Li Z, Sun J, Wang Q, Lin J, Ye H, Liu F, Srinivas S, Li D, Zhu B, Liu YH, Tian GB, Feng Y. 2016. Dissemination and mechanism for the MCR-1 colistin resistance. PLoS Pathog 12:e1005957.

11.	Xu Y, Wei W, Lei S, Lin J, Srinivas S, Feng Y. 2018. An evolutionarily conserved mechanism for intrinsic and transferable polymyxin resistance. mBio 9:e02317-17.

12.	Liu YY, Chandler CE, Leung LM, McElheny CL, Mettus RT, Shanks RM, Liu JH, Goodlett DR, Ernst RK, Doi Y. 2017. Structural modification of lipopolysaccharide conferred by *mcr-1* in Gram-negative ESKAPE pathogens. Antimicrob Agents Chemother 61:pii: e00580-17.
